# LoRa Propagation and Coverage Measurements in Underground Potash Salt Room-and-Pillar Mines

**DOI:** 10.3390/s25123594

**Published:** 2025-06-07

**Authors:** Marius Theissen, Amir Kianfar, Elisabeth Clausen

**Affiliations:** Institute for Advanced Mining Technologies (AMT), RWTH Aachen University, 52062 Aachen, Germany; akianfar@amt.rwth-aachen.de (A.K.); eclausen@amt.rwth-aachen.de (E.C.)

**Keywords:** LoRa, underground, mining, room and pillar, network, propagation

## Abstract

The advent of digital mining has become a tangible reality in recent years. This digital evolution requires a predictive understanding of key elements, particularly considering the reliable communication infrastructures needed for autonomous machines. The LoRa technology and its underground propagation behavior can make an important contribution to this digitalization. Since LoRa operates with a high signal budget and long ranges in sub-GHz frequencies, its behavior is very promising for underground sensor networks. The aim of the development and series of measurements was to observe LoRa’s applicability and propagation behavior in active salt mines and to detect and identify effects arising from the special environment. The propagation of LoRa was measured via packet loss and signal strength in line-of-sight and non-line-of-sight configurations over entire mining sections. The aim was to analyze the performance of LoRa at the macroscopic level. LoRa operated at 868 MHz in the free band, and units were equipped with omni-directional antennas. The K+S Group’s active salt and potash mine Werra, Germany, was kindly opened as a distinctive experimental setting. The LoRa exhibited characteristics that were highly distinctive in this environment. The presence of the massive salt allowed the signal to bounce along drift edges with near-perfect reflection, which enabled travel over kilometers due to a waveguide-like effect. A packet loss of below 15% showed that LoRa communication was possible over distances exceeding 1000 m with no line-of-sight in room-and-pillar structures. Measured differences of Δ50dBm values confirmed consistent path loss across different materials and tunnel geometries. This effect occurs due to the physical structure of the mining drifts, facilitating the containment and direction of signals, minimizing losses during propagation. Further modeling and measurements are of great interest, as they indicate that LoRa can achieve even better outcomes underground than in urban or indoor environments, as this waveguide effect has been consistently observed.

## 1. Introduction

The present paper aims to investigate signal propagation within communication networks in underground mines, focusing on specialized LoRa networks to support future digitalization efforts for mining operations. The NEXGEN SIMS project had a focus on developing autonomous carbon-neutral mining processes [1]. Advanced communication systems formed one cornerstone of the project, encompassing several parallel initiatives. Communication networks in general serve as a fundamental enabler for the process of digitization, as well as a multitude of related use cases, including automation, localization, and the relaying of machine and operational data. Nevertheless, a comprehensive grasp of behavioral patterns and anticipated performance outcomes is indispensable prior to the feasibility of a full installation on-site. Especially in the former use case, LoRa technology is a promising solution for sub-GHz communication and has demonstrated its effectiveness in urban settings but remains underexplored in mining environments.

While network propagation is an active area of research, underground mines are rarely studied within this context. Developing specialized networks is crucial to address the dynamic and challenging conditions found in underground mines. This paper fills this research gap by presenting a measurement concept based on established methods, ensuring comparability and reproducibility. Measurements were conducted in real, active mining environments. As a partner in the project, the K+S group enabled the tests to be performed in the salt and potash mine Werra in Germany. Werra has a room-and-pillar mine structure and thus shows a particularly topologically exciting environment with long drifts in a constantly crossing chessboard-like arrangement. This made it possible to examine particularly long “straight” stretches and sharp edges for propagation influences. The influence of the surrounding material of solid salt was also a novel aspect. These are contrasting conditions to those found in urban, rural or even indoor scenarios. Nevertheless, use cases are of the same nature if one wishes to centrally collect information such as the monitoring of machines in operation or environmental controls for optimization and safety enhancement.

Hence, both signal and environmental parameters for thorough analysis were collected. The evaluation of the findings provides a groundwork for the future modeling and interpretation of signal behavior in underground mines.

Mining faces challenges such as hazardous working conditions, resource inefficiency, and high operational costs. Digital technologies aim to address these issues by providing safer environments, optimizing resource use, and reducing costs.

Mining as a unique environment presents distinctive conditions that are not necessarily negative for establishing a potential network. These conditions can significantly influence the specific topology of the mine itself, which is largely determined by the extraction methods employed. The method is closely tied to the mineralogy of the site, which is in itself important for any propagation. Additionally, there are generalized dynamics at play, stemming from both corporeal and radial influences due to the operational activities of personnel and machinery on site.

Therefore, advancements like IoT-based sensor networks, autonomous mining vehicles, and real-time monitoring systems have created a demand for robust communication systems to ensure seamless data transfer and system integration.

Planning these layouts requires accurate models based on robust propagation data. Understanding signal behavior in complex underground environments is vital for addressing challenges like geological formations. Using real-world propagation data from active mining sites, engineers can develop reliable models to guide design decisions.

To summarize, this paper aims to provide a novel measurement series on LoRa propagation in underground room-and-pillar mines, addressing an identified research gap in this field. The remainder of this paper is organized as follows:

Section 2 reviews previous experiments on underground LoRa communication and identifies key gaps in current research.

Section 3 outlines the theoretical physics framework used.

Section 4 introduces and expands on foundational concepts required for communication and propagation analysis.

Section 5 describes the proposed measurement principles.

Section 6 presents the measurement setup chosen for this work.

Section 7 lists and describes the hardware components involved.

Section 8 discusses in detail the results of the measurement campaign conducted at Werra, Germany, and explains their implications.

Section 9 analyzes the measurements performed near Kvarntorp, Sweden, and compares them with the results obtained in Werra.

Finally, Section 10 offers concluding remarks and highlights the main findings of the study.

Appendix A has been added to provide raw measurement data.

## 2. Related Work

This paper aims to position its contributions within the broader context of scientific research on wireless communication in underground environments. In particular, it contrasts the presented work with related studies focusing on LoRa propagation in subsurface settings. While several previous publications have explored the feasibility and performance of LoRa communication underground, many of them have been conducted in controlled test environments or abandoned tunnels [2,3].

In contrast, this study emphasizes real-world measurements in actively operated mining environments, where signals encounter dynamic factors such as machinery, ventilation systems, and irregular geometries. By comparing shared characteristics and research goals with existing studies, this paper highlights both the common ground and the key distinctions that underline the novelty of this approach. The focus on operational mines, extended measurement distances, and context-specific interpretation of the results sets this work apart and contributes new insights into the applicability and limitations of LoRa-based communication in complex underground conditions.

Several foundational studies form the basis for understanding radio frequency propagation in underground environments. While these publications are somewhat dated, they remain highly relevant due to their comprehensive experimental set-ups and continued applicability in comparative analyses. Notable among them is the work by Zhou et al., which presents an extensive measurement campaign across a wide frequency range—from 455 MHz to 5800 MHz [3]. Their results have served as a reference point for many subsequent studies. Another frequently cited publication is by Hakem et al., which investigated signal behavior at 2.4, 5.8, and 60 GHz. The authors observed a clear trend of increasing path loss with increasing frequency, a phenomenon that has significant implications for underground communication system design [4].

Before reviewing more recent publications that specifically examine LoRa-based systems in such environments, an important caveat must be addressed. The growing number of LoRa-related studies is certainly beneficial, as it reflects the expanding interest in and applicability of this low-power communication technology. However, the surge in recent publications has also led to a terminological fragmentation. Inconsistent use of key terms and definitions has complicated direct comparisons and often obscured the underlying contributions of individual studies.

Against this backdrop, it becomes essential to clarify the methodological and contextual distinctions between related works. In the following section, we examine selected publications that focus specifically on LoRa propagation in actively operated underground mining environments in order to highlight both shared experimental characteristics and the unique aspects of the presented approach.

In the context of this paper, the term “underground” refers specifically to mining environments located at a considerable depth below the surface, consistent with the definition used in the field of subsurface resource extraction. However, in several related publications, the term “underground” is employed in different ways, often referring to shallow-buried sensors located just beneath the surface. Examples of this broader usage include studies such as [5,6,7], where the measurement set-ups involve near-surface installations rather than deep mining drifts.

Other works extend the term “underground” to refer to urban infrastructure, such as drainage systems [8] or soil and pipeline monitoring scenarios [9]. While these studies may share certain characteristics with underground mining research—such as non-line-of-sight conditions or limited accessibility—they differ significantly in geological composition, structural constraints, and electromagnetic behavior.

In this context, the term Wireless Underground Sensor Networks (WUSNs) is also frequently used, but it does not necessarily imply deployment in underground mining environments. Instead, it often refers to sensors shallowly buried in soil, typically within a depth of less than one meter.

This observation is not intended as a criticism but rather as a clarification, since such terminological overlap can lead to confusion during literature reviews. Comparative analysis with these works is limited in relevance, except in terms of general LoRa transceiver settings or hardware configurations.

Unlike the above-mentioned studies, in the present work, the devices are installed in open configurations within enclosed, thick-walled mining spaces, surrounded by highly attenuating geological materials. By contrast, the previously cited works often examine semi-permeable or open environments, where propagation is affected by only minimal layers of soil or construction material.

These are, in a manner of speaking, “related works with no relation”—studies that share the keyword “underground” but diverge significantly in scope, setting, and scientific relevance to underground mining. Once these are filtered out, the number of truly comparable studies focusing on LoRa communication in actively operated underground mines becomes significantly smaller.

Among the most prominent contributions to the study of LoRa propagation in active underground mining environments are the works by P. Branch [10,11], which investigate LoRa communication at 915 MHz in Australian gold mines. These studies introduced several foundational models for LoRa signal behavior in mining scenarios, particularly focusing on the block caving mining method. Reflections and diffraction phenomena were examined in specific sections of the mine drifts. However, the measurement scope in these studies was limited to selected drift segments and did not cover large-scale or full-area propagation analysis.

Another relevant example is provided by Abrardo et al. [2], who conducted measurements in the context of monitoring medieval aqueducts in Italy. Although not considering a mining environment, the work contributes to understanding LoRa performance in highly attenuating underground conditions, noting strong signal losses due to dense stone structures.

What distinguishes the present paper from these previous works is its explicit focus on area-wide propagation in mining environments, particularly across multiple directional turns within structurally regular room-and-pillar geometries. Rather than focusing on isolated drift segments, this study analyzes signal coverage across broad sections of the mines. Furthermore, by conducting measurements in two operational mines with differing mineralogical compositions, the dataset allows for an expanded evaluation of environmental influence parameters. These results contribute to a more systematic understanding of underground LoRa propagation, indicating that mineral composition, while relevant, plays a lesser role than factors such as topology and wall roughness.

## 3. Propagation Concepts for the Microscopic Scales

At the microscopic level, this study deals with influence factors, which are dimensionally below the wavelength of approximately 30 cm. Alternatively, one can define this level as including effects stemming from the particle–wave duality in electromagnetism. Electromagnetic radiation and propagation theory will be used to study the unique environment of the mining area. Hence, a clear understanding of the fundamental concepts and phenomena that form the basis of all discussions in the scientific literature is required.

In these sections, the problem of propagation is considered in two stages. First, the basics of microscopic effects and mathematical backgrounds are outlined. From these individual effects, the macroscopic phenomena are defined and summarized as given in the respective literature. This allows them to be placed in a context and physical scope.

### 3.1. Maxwell Equations

The Maxwell equations form the basis of all the descriptions, models and effects required. They describe how electric and magnetic fields, as well as electric charges and electric currents, are related under boundary conditions. Maxwell’s equations can be given in several forms, all of which are equivalent, but for the sake of clarity, the most common form of the equations is used here—the partial differential form in SI units and as four separate equations.

(1) $$\begin{array}{cc} \nabla \cdot E & =\frac{\rho }{\epsilon_{0}}\\ \nabla \cdot B & =0\\ \nabla \times E & =-\frac{\partial B}{\partial t}\\ \nabla \times B & =\mu_{0}\left(J+\epsilon_{0}\frac{\partial E}{\partial t}\right) \end{array}$$

Important mathematical concepts for understanding these equations are vector analysis [12] with consideration of vector products and scalar products, as well as partial derivatives that extend over space and also over time. Topics include partial differential equations [13] and classical electrodynamics [14].

### 3.2. Scattering

In physics, scattering refers to a variety of processes in which moving particles or radiation, such as light or sound, is deflected from a straight path due to localized irregularities in the medium traversed, which includes dust, other particles and even radiation itself. A comprehensive overview of the theoretical physical description of classical electromagnetic radiation scattering can be found in many textbooks such as [15]. Scattering can occur on all scales, from single electrons to molecules to imagined solid slabs, but fundamentally, it is the same process. When an obstruction is exposed to an electromagnetic wave, the electrical charges within the obstruction are caused to oscillate by the wave’s electric field, as described in Equation (1). Accelerated electric charges emit electromagnetic energy in all directions, and this emission is then referred to as the radiation scattered by the obstacle.

Although for more precise calculations, several different modeling approaches must be chosen to adapt the radiation to the properties of the obstacle, it is sufficient, as a basic model for scattering at a solid wall or pillar in a mine, to reduce to a simpler model. This model describes, in a first approximation, the scattering of electromagnetic radiation at a solid “Plane Boundary” with specific properties and, most importantly, dimensions.

**Figure 1:**
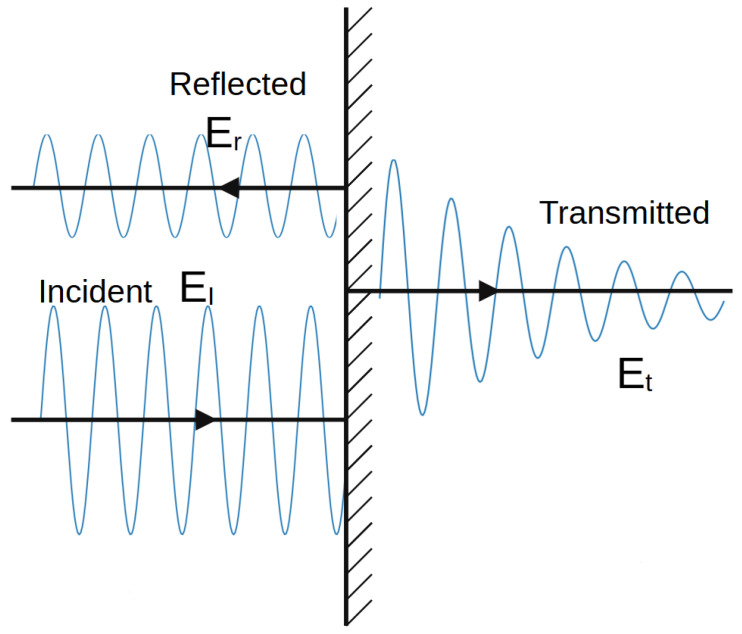
Reflection and transmission at a plane boundary. $E_{I}$ is the incident/incoming wave, $E_{r}$ is the reflected wave component, and $E_{t}$ is transmitted. This graphic is based on/inspired by a graphic found in [14] and many more works.

When considering the Maxwell Equations (1), the first solution that can be used for the set-up depicted in Figure 1 is the so-called plane wave. It is the complex representation using the exponential function, reading

(2) $$E_{t}\exp\left[i\omega \left(\frac{N_{1}z}{c}-t\right)\right]\;\left(z>0\right),$$

and

(3) $$E_{i}\exp\left[i\omega \left(\frac{N_{2}z}{c}-t\right)\right]+E_{r}\exp\left[-i\omega \left(\frac{N_{2}z}{c}+t\right)\right]\;\left(z<0\right).$$

Equation (3) shows the two separate waves, namely the reflected part and the incident wave. The boundary is at z=0.

This is a basic model to describe scattering [15]. Even though it represents the most basic level, it can already capture many of the more complex aspects, such as phase differences, the separation of the system into components with individual waves, and superposition.

The question arises as to how applicable this model might be in underground environments. The primary argument for keeping this model in mind is the relationship between the wavelength and the surrounding environment. LoRa, operating with sub-GHz frequencies, is stable in the presence of dust, and its wavelength is in the range of 20 to 30 cm. This is comparable to the structure of the surrounding surfaces, where even a simple flat surface cannot be neglected in a first-order approximation.

### 3.3. Refraction

When light or an EM wave in general moves from one medium to another, it changes direction; i.e., it is refracted. The refractive index is the ratio of the speed of an electromagnetic wave in a vacuum *c* to the speed in the medium. Assuming an incidence angle to the surface normal of $\theta_{1}$, the refraction angle $\theta_{2}$ can be calculated from Snell’s law:

(4) $$n_{1}\sin\theta_{1}=n_{2}\sin\theta_{2}.$$

**Figure 2:**
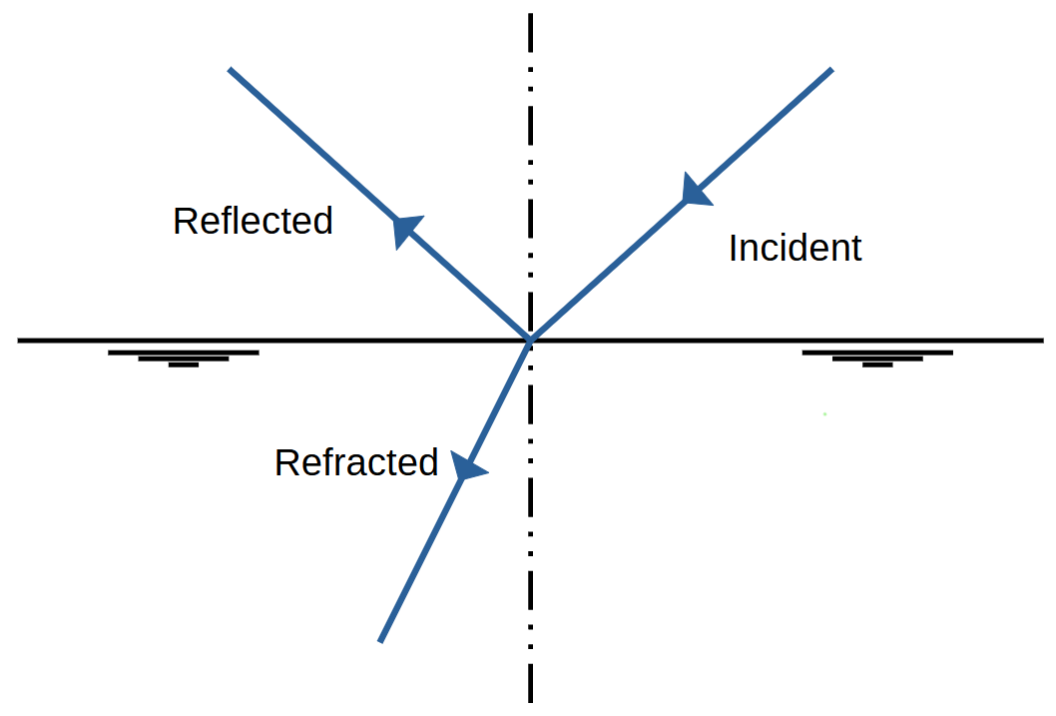
Reflection and refraction.

When the approximated beam enters a material with a higher refractive index, the angle of refraction will be smaller than the angle of incidence, and the ray will be refracted towards the normal of the surface. The higher the refractive index, the closer to the normal direction the ray will travel. When passing into a medium with a lower refractive index, the ray will instead be refracted away from the normal, towards the surface. Snell’s law Equation (4) (also known as the Snell–Descartes law, the ibn-Sahl law, and the law of refraction, as it was rediscovered multiple times) is a formula used to describe the relationship between the angles of incidence and refraction, when referring to light or other waves passing through a boundary between two different isotropic media, such as water, glass, or air; see Figure 2. In optics, the law is used in ray tracing to compute the angles of incidence or refraction [16] and in experimental optics to find the refractive index of a material [17].

### 3.4. Diffraction

If a wave is deflected by an obstacle, it is called diffraction. This allows waves to propagate through areas that would be blocked on a straight line. According to the Huygens–Fresnel principle, diffraction is caused by a new wave formation along a wave front. These new waves can combine constructive and destructive to produce interference patterns. This combination is called interference and means that, acting via the superposition principle, waves’ valleys add up, thus showing an amplifying effect. In the case of destructive interference, a valley adds to a mountain and thus reduces it to zero. The relative distance or position of these valleys or hills is called the phase of the wave, and a difference between waves is then called a phase shift, as the wave pattern repeats; see [17] for a basic introduction on the principle of diffraction.

### 3.5. Transmission and Penetration

When an EM wave hits an object, the wave description is divided into several parts. Wave components before the interaction are labeled incident or incoming waves. At the object boundary on which the wave strikes, there is a part that is reflected, i.e., that propagates against the direction of the incident wave. If one now considers an object of finite diameter, there are inner waves that scatter inside the medium, i.e., internal waves. Everything that emerges from the finite object has been transmitted. This action with finite objects describes the passing through or penetration of the object by the wave [15].

### 3.6. Absorbtion

Absorption is inferred from Section 3.2. Absorption is the loss when the intensities of the reflected wave components are offset against the transmitted wave components compared to the incident wave [15]. It is possible due to various effects and interactions, where parts of the spectrum, intensity, or simply energy are transferred to the medium of the transmitted wave. For example, microwave ovens operate on this principle by functioning at a resonance frequency of water molecules to induce vibrations and, therefore, heat, which occurs around 2.4 GHz. However, these effects cannot be analyzed in detail at this point, as their foundations lie in material physics and require fundamental knowledge of quantum mechanics, which is not the focus of this investigation.

### 3.7. Shadowing

In most applications, obstacles stand between transmitting and receiving antennas. They can vastly differ in their density and influence on the specific signals. One can think of open outdoor areas as having very few obstacles, while indoor environments are often filled with them. This variability in the environment is accounted for by modeling the density of obstacles and their absorption characteristics as random variables. This process is known as shadowing [18]. Shadow fading occurs over a duration, lasting several seconds or minutes. These points and further explanations can be found in [19].

## 4. Propagation Concepts for the Macroscopic Scales

In order to facilitate a more comprehensive discussion of the fundamental components of an electromagnetic wave and the classification of its propagation effects, it is first necessary to provide a basic introduction to the topic and an overview of the propagation phenomena. In the context of communication, the term channel is typically employed to describe the medium through which information is transmitted. Since the ether has been dismissed as a medium for electromagnetic waves as a result of the Michelson–Morley experiment [20], the term has been used in the sense of the frequency range and usually also the protocol layer chosen for information transmission. The use of these channels is subject to various effects and influences that can be attributed to the signal and, in general, to the transmission behavior. As succinctly outlined in the work “Propagation Modelling for Wireless Communication” by Indrakshi Dey [18], there are six distinct phenomena that warrant assessment for applicability in the underground mining environment.

Therefore, the structure of this specific part of Indrakshi Dey’s work [18] is also adopted in the following sections.

These phenomena describe the manner in which effects such as shadowing, diffraction, and scattering occur. Shadowing is caused by obstacles of a size much larger than the propagating wavelength [18]. This can be attributed to a multitude of factors, including humans or machinery, but also extends to the presence of dust particles or water droplets at higher frequencies [18]. Diffraction is a phenomenon that arises from the bending of traveling waves around an obstacle [17], accompanied by a charge change impact loss due to the variation in relative distance between the transmitter and the receiver. In addition to diffraction, one must also consider scattering, which occurs via the interaction of the traveling waves or any modes of communication with objects of dimensions on the order of the wavelength or less; see Section 3.2. These wave interaction effects must be considered independently, as scattering and diffraction can occur at the same object with the same communication option if it has a wide enough channel selection, such as that offered by 5G [21]. Here, the two regimes of wave interaction can happen simultaneously. Understanding the combined effects of diffraction and scattering is essential for characterizing the dynamic behavior of a communication channel. These effects, along with other wave phenomena, contribute significantly to spatio-temporal variations observed in wireless environments.

### 4.1. Spatio-Temporal Variation of the Channel

According to [18], it is a well-established fact that propagating waves and structures result in reflection, diffraction, scattering, and refraction, resulting in losses beyond the standard, as given in Equation (5). Fading and shadowing are grouped together as umbrella terms. When a transmitted signal experiences fading and shadowing, both its amplitude and phase fluctuate over time [18]. This means spatio-temporal variation of the channel, where signal components are randomly delayed, reflected, scattered, and diffracted, leads to combinations of the waves in constructive and/or destructive ways; see Section 3.4. The results are short-term signal variations known as multipath fading [18]. When mean signal levels vary over a larger time frame and structures or objects are identified as the cause, one speaks of shadowing [18].

Spatio-temporal variations of the signal parameters are given by [18]:

- Distribution of Arrival Time Sequence;
- Distribution of Path Amplitudes;
- Distribution of Path Phases;
- Interdependence of Path Variables.

This will prove to be a significant point of discussion in the context of underground mining, which is highly diverse and changing in topological characteristic. This along side its specific material surroundings and heavy machinery density, makes it unique in terms of spatio-temporal variation. However, the concept of spatio-temporal variation is particularly broad in terms of identifying the specific structures responsible for the observed changes in the channel.

### 4.2. Temporal Variation of the Channel

The propagation environment can influence the channel in a non-stationary manner [18]. Relative motion or repositioning of the transmitter or receiver can have large influences alongside movement of the surrounding objects [18]. However, most experimental measurements in the literature consider the communication channel to be stationary [18], which is highly unrealistic for mining environments over both long and short periods. Over longer periods, the shape and 3D geometry of the mining site changes and expands [22]. On a timescale of a few minutes, large machinery and vehicles can block entire drifts or network nodes, resulting in significant shadowing.

The assumption of stationarity, however, remains valid for the measurements conducted in this series, as it was specifically designed in the measurement plan with the intention of reducing and eliminating its contribution to the propagation behavior.

### 4.3. Large-Scale Path Loss

Specifications of a channel can vary in single-input–single-output (SISO) systems due to an increased distance between the transmitting and receiving antenna, or in multi-input–multi-output (MIMO) systems due to greater antenna spacing [18]. The EM wave is subject to spread, resulting in path loss in free space or even in a vacuum. This effect is called the Free-Space Path Loss and is described by the Friis transmission equation with a logarithmic dependence [23]. It reads in the antenna gain focused representation as

(5) $$\frac{P_{r}}{P_{t}}=G_{t}G_{r}{\left(\frac{\lambda }{4\pi d}\right)}^{2},$$

where $P_{t}$ is the power into the transmitter, $P_{r}$ is available power to the receiver, $G_{t},G_{r}$ is the transmitter and receiver gain, λ is the wavelength as usual, and *d* is the distance between the two units. To make the equation more field- and engineering-applicable, it can be formulated using *decibels* without loss of information. It reads

(6) $$P_{r}^{\left[\mathrm{dB}\right]}=P_{t}^{\left[\mathrm{dB}\right]}+G_{t}^{\left[\mathrm{dBi}\right]}+G_{r}^{\left[\mathrm{dBi}\right]}+20\log_{10}\left(\frac{\lambda }{4\pi d}\right).$$

A large distance makes the communication channel vulnerable to more obstacles. This environmental path loss can be defined as the attenuation of the received signal due to these channel variations [18]. In an indoor environment, path loss is also facilitated by absorption losses through materials and minerals [18].

Given that LoRa is distinguished by its extensive range [24], this is a particularly crucial case for elucidating the mechanisms of propagation. In underground environments, the interaction with a multitude of minerals and at varying strengths represents a significant departure from the models and results observed in urban or industrial settings.

### 4.4. Delay Spread

One reason for a delay spread is multipath fading [18]. It results in multiple copies of the transmitted signal reaching the receiver at different points in time [18]. It is possible that the various components of the signal will not arrive simultaneously [18]. The temporal domain representation of the original signal is referred to as the delay spread [18]. Should the period of the baseband signal surpass the delay spread, the outcome will be intersymbol interference (ISI) [18]. The mean excess delay is the measure of the average delay spread caused by multipath components [18]. Root-mean-square (RMS) delay spread is defined as the standard deviation of the delay of reflections, with the weighting proportional to the energy in the reflected wave [18].

The measurements demonstrate that, due to the substantial mineral walls, the concept of multipathing and copies is of greater significance and drastically more pronounced in underground mining compared to open field tests or even industrial settings.

### 4.5. Frequency Dependence of Channel Statistics

The wireless channel characteristics may undergo significant alterations in response to changes in the frequency of operation [18]. It should be noted that this variation is not necessarily proportional, as resonances in the environment can occur [18]. The statistical characteristics of a wireless channel can vary considerably in different environments, with changes occurring at different frequencies of operation [18]. The extent of path loss and continuous wave penetration loss is contingent upon the composition of the surrounding materials and minerals [18]. The delay spread or the power delay profile decay exponent varies with environmental properties [18].

The phenomenon of resonance can be considered the primary reason for the transition from diffraction to scattering in the case of corpuscles of dust, water droplets, and similar environmental factors [15]. It is important to consider this when working in dusty environments, as dust can block the signal. One might consider machinery, which is dependent on 5G connectivity. However, the work carried out at the mining face produces interfering dust, which has the potential to disrupt the signal.

### 4.6. Noise and Co-Channel Interference

The presence of interference, noise, inter-symbol interference (ISI), and intermodulation can all have a detrimental impact on the performance of a system [18]. The advent of multiple-input–multiple-output (MIMO) systems and the electromagnetic reuse of the radio spectrum has led to the emergence of co-channel interference [18]. When two transmitters operate at identical carrier frequencies, co-channel interference is inevitable [18]. It is possible that both the desired signal and the interfering signal may travel to the receiver via the same or different paths [18]. In the event that the signals traverse different paths, they will fade independently, regardless of whether the distributions are identical or not [18]. The degree of co-channel interference is quantified by the signal-to-interference-plus-noise ratio (SINR) [18].

In general, the underground mining sector only has their own signal generators due to the shielding, surrounding material. In urban settings, one must work with a multitude of foreign anisotropic sources of radiation and channel noise [18].

### 4.7. Summary of the Propagation Concepts

What follows is a brief summary of the most important concepts in the description and analysis of propagation of electromagnetic radiation.

There are two fundamental levels. They differ in terms of orders of magnitude in their operational length dimension and the derivability of results from direct physical formulas and principles. The concepts at the microscopic scale are based on Maxwell’s equations and the first optical principles, which have been rediscovered multiple times over a thousand years. These microscopic concepts, such as scattering, refraction, and diffraction, address the deviation of electromagnetic waves from linear propagation due to interaction with surfaces or matter in general.

Furthermore, it was important to clarify the topics of transmission, penetration, absorption, and shadowing. These concepts refer to losses in measurable radiation compared to free propagation when measurements are taken at specific points within a set-up. All these fundamental and well-known observations are central to understanding how an electromagnetic wave, when considered in isolation within an otherwise pure environment, can interact with components that describe physical phenomena. These influences are always present unless they are intentionally reduced in laboratory set-ups.

The concepts at the macroscopic scale primarily consist of overarching influences that can occur in communication networks. They are often considered over large areas that define the general range of radiation for network set-ups. When operating in a low-radiation environment and introducing relevant signals into this measurement system, some of these concepts quickly fade into the background. Additionally, these effects are rather dependent on resonances, such as co-channel or channel statistics. Depending on the type of radiation being considered, these effects can be minimized.

## 5. Measurement Parameter Selection

This theoretical discussion shall be followed by considerations on how a complex system such as signal propagation can be effectively measured in real time in the field. This section outlines the rationale behind the selected measurement parameters and the practical set-up used for capturing signal-propagation characteristics in real-time field conditions. The primary purpose of the transmitted signals is to establish and maintain a network connection. A network is fundamentally a system that enables the exchange of data between distributed endpoints [18]. Therefore, analyzing a network or a transmission path that describes the smallest element in a network of many possible points is to be based on the characteristics of a network. To name just a few, at this point, it should be noted that the characteristics of a network are mostly related to the expected performance of the endpoints with the incoming data. If a task at the endpoint expects or requires large data packets with low latency, the network must be tested, optimized, and installed for these properties.

Each measurement requires a careful preliminary decision on the necessary, appropriate, and target parameters to be measured. In signal propagation there are a variety of selection options, but the most common are dBm, IDs, packetloss and a selection of environmental properties [18,19].

Starting with dBm, it is one of the most widely utilized and essential units in radio and signal analysis. dBm, short for decibel-milliwatts, represents power levels on a logarithmic decibel (dB) scale relative to one milliwatt (mW). This unit is frequently used to quantify transmission power because it efficiently expresses both very large and very small values in a compact form through the log scale. A power *P* in mW written as *x* in dBm is given in [25] and reads

(7) $$x=10\log_{10}\frac{P}{1\;\mathrm{mW}}.$$

The dBm is a fundamental measurement that provides information about the strength of the field at a given point in space-time and an indicator for the influences along the path the signal has taken. At the most basic level, it is often used to test how “good” the signal is compared to the power being emitted.

However, this general statement about the signal should not be made directly on the basis of the dBm value alone. As a rule, other application-related values should be recorded. These include the packet loss. In measurement set-ups, a sequence of *n* different clusters of information is sent to one measurement point. The measurement can now include how many of these clusters have been successfully recovered and decoded. This is averaged influence information for the path taken by the signal. It is often assumed that dBm and packet loss are related. This is only true in very flat and static areas. For example, radio pulsars here on Earth are distinguished by tiny dBm values [26]. But each pulse can be measured very precisely. Of course, one can also take a more common network example where, in a crowded area, the signal strength itself is very strong, but due to multi-user access or the use of the same frequency, the packets cannot be sent at the correct interval or are mixed up between users and therefore cannot be decoded.

Some environmental parameters can be used to gain insight into the location and possible reasons for the measured values in dBm packet loss. Yet, as these parameters only give absolute results, context is needed to find explanations for the values. It is clear that position and/or time must be recorded, as networks are area-related concepts. One set of environmental influencing factors can be temperature and humidity variations when operating at near-resonant frequencies of water or surrounding dust particles.

## 6. Conceptual Measurement Set-Up

With the measurement parameters specified, the set-up concept will be explained in the following section [27]. The room-and-pillar method is an old and widely used mining technique [22]. It is ideally suited for deposits that occur in horizontal seams, such as coal, potash, salt, or limestone, among others. These seams typically range in thickness from low to medium. The method creates an underground structure resembling a chessboard, formed by the leftover standing material, which is strategically retained to provide support. From a wireless network point of view, massive and angular obstacles are not only common but essential to the environment; they make up the entire environment in the strict sense. Therefore, in the two-dimensional view from above, several scenarios have been included to show the behavior of the propagation of EM waves. Without going into more detail about room-and-pillar mining as a method, the most important characteristics that give shape to the structure should be mentioned. A very detailed source of information on this method can be found in [22].

### 6.1. LoS Set-Up

**Figure 3:**
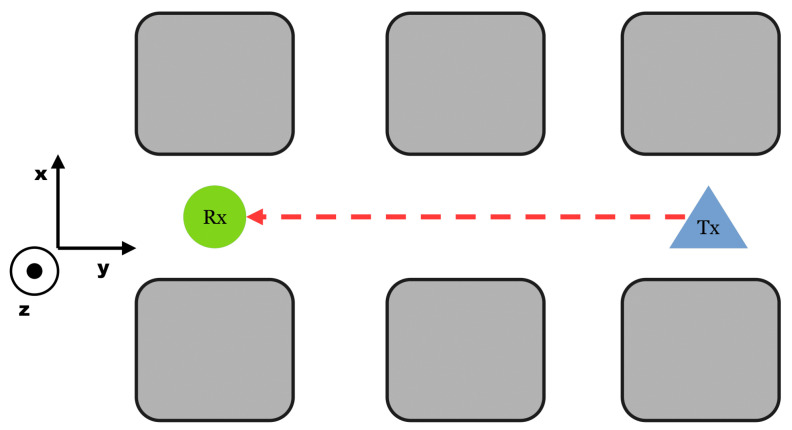
Set-Up LineOfSight. The transmitter “Tx” can be seen or connected to the receiver “Rx” over an imaginary straight line. This is often associated with the first-order Taylor expansion for waves [18].

The “LineOfSight” (LoS) set-up is, as the name implies, a configuration where the transmitter “Tx” can be seen or connected to the receiver “Rx” over a straight line; see Figure 3.

### 6.2. Near LoS

**Figure 4:**
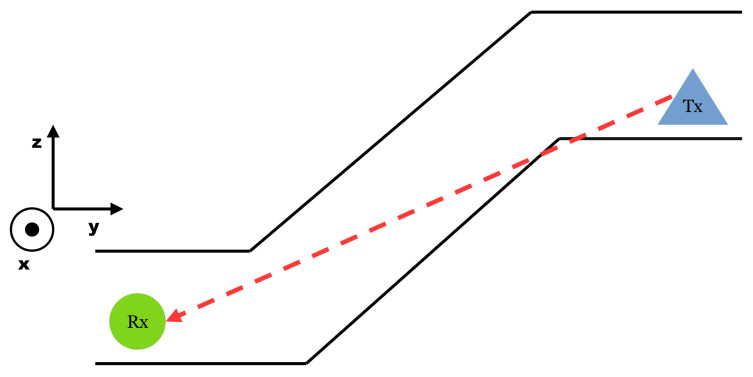
Set Up Near LineOfSight. It works on the principle that the LineOfSight is interrupted in the straight line graph, but only so much as influences like wave polarization in the *x*, *y* or *z* dimensions may even be larger than an obstruction.

Near LoS is a step away from a clear line to connect the two points, Tx and Rx. In an underground mine, it is not a given to have absolutely even elevation. While the roof height varies, the drift itself can also twist up and down. It follows the structure of the deposit. For this reason, even if the top-down view on the map implies LoS, it can be limited in one dimension, often on the *z*-axis; see Figure 4.

### 6.3. NLoS Set-Up

**Figure 5:**
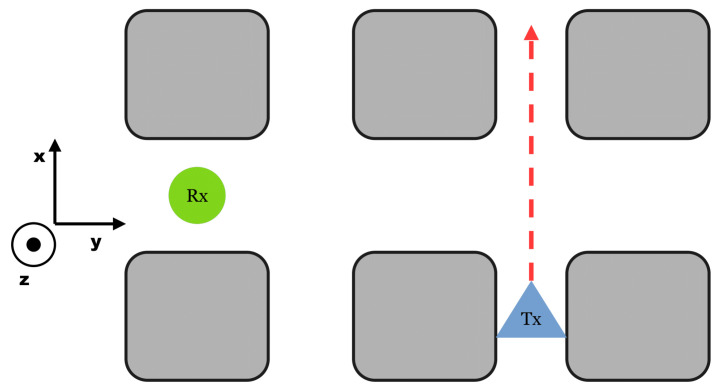
Set Up for No LineOfSight.

If all three space dimensions are obstructed, it is called “No LineOfSight” or NLoS. The units are not able to connect via straight pathways. Other effects like penetration or reflection must occur for networking; see Figure 5.

### 6.4. Long-Range Set-Up

To distinguish from the area or section-wide set-up tests, another set-up was used. The Long Range set-up is a transition from LoS to Near LoS to almost NLoS over a long and mostly straight route in two dimensions. The elevation changes make it almost instant near LoS, and over a long distance, any straight line would pass through hundreds of meters of solid material. Although the principle is very similar to the conceived set-up described in Section 6.2, it has specific and unique properties.

The long-range set-up is intended to check whether ranges occur aboveground and thus have an initial justification for the transferability of assumptions. Furthermore, several effects can only build up a measurable influence through longer distances, including reflections, scattering on corpuscles, and others. These individual effects, which are often characterized by smaller contributions, can then be better recorded and then also reconstructed if they appear statistically more often with distance length and can thus be measured variably.

### 6.5. Configurations for Any Set-Up

To allow easier comparison between all set-ups, the following configurations were kept constant.

**Figure 6:**
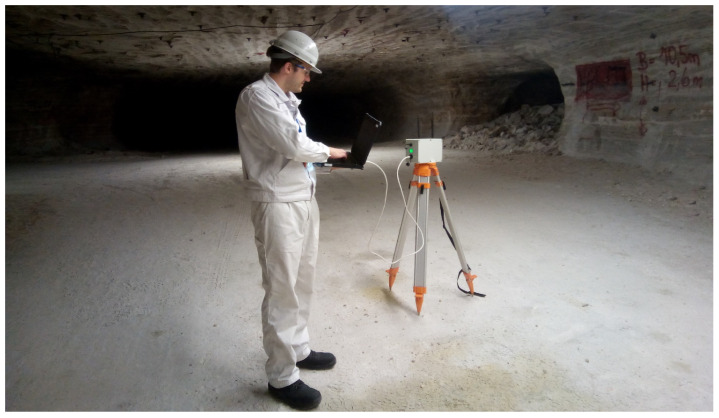
LoRa field test configuration. This image shows the set-up and launch configuration of the Alpha station in preparation within the test area by a scientific research assistant. Note the positioning at a height of approximately 1.60 m using a tripod and its placement in the middle of the drift. This configuration is intended for an LoS measurement using the implied x and y coordinates. However, due to the varying height and inclination of the drift itself, the classic definition of LoS across three coordinate axes is not met at several points.

- Height: Both units were placed on a tripod at a height of 1.6 m or held at this level manually; see Figure 6.
- Movement: The units were carried or mounted temporarily outside a truck without being blocked by the vehicle itself.

## 7. Hardware and Environment Description

This section describes the test apparatus and the mine in which it was used. For this purpose, the unit called the Walpurga unit is first described in more detail, and then the Werra mine is described with important characteristics for the experiment.

### 7.1. Walpurga Unit

The Walpurga unit was designed to be a multi-network-capable development unit suitable for both underground and open-pit mining environments. The IP 66 rating was chosen to ensure dust and water resistance. The unit incorporated a 26,800 mAh powerbank for the Raspberry Pi 4 and the LoPy 4 board as the main computing and software deployment elements. Important connections were extended to sealable ports on the outside of the enclosure, such as Ethernet, power over USB to charge the power bank, and the power switch itself. The units were either placed on a tripod at a height of 1.6 m or carried.

The LoRa signal was transmitted using an omnidirectional antenna alongside a GPS antenna, which was turned off for the tests conducted.

The set-up had the following characteristics and parameters:

- **LoRa board:** LoPy 4 by pycom;
- **LoRa expansion shield:** Pytrack 2.0X by pycom;
- **Main board:** Raspberry Pi 4;
- **Signal frequency:** 868 MHz;
- **Spreading factor:** 7;
- **Bandwidth:** 125 KHz;
- **Coding Rate:** 4/5;
- **Transmitter Power Output:** +14 dBm;
- **Signal modulation:** Chirp-Spread-Spectrum;
- **Payload Lenght:** 8 bytes;
- **Number of packets per test:** 50;
- **Power bank:** 26,800 mAh;
- **Housing:** Dust and water resistant.

### 7.2. Mine Environment, Werra

The measurements were first conducted in actively used and constantly expanding sections of the Werra mine of the K+S Group. This is a room-and-pillar mine in which salt and potassium salt are extracted. Excavation is performed by drilling and blasting. Possible influences on signals are mentioned in the topological discussion of the signal measurement.

**Figure 7:**
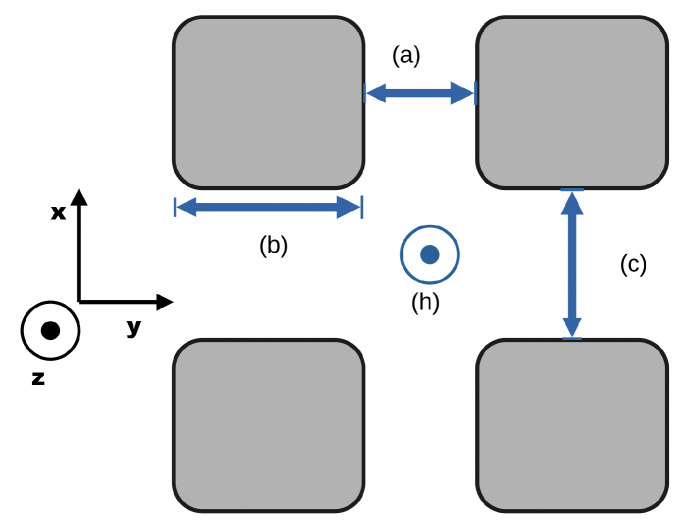
Sketch mining cross-section for a room-and-pillar mine. For reference, the dimensions associated with the Werra mine, Germany, are given. (a) ≈ 20 m, (b) ≈ 50 m, (c) ≈ 25 m, (h) ≈ 5 m.

Approximate values for the minimal cell of the repeating mining cross-section are given in Figure 7.

## 8. Discussion on Propagation Behavior in the Potash Salt Mine Werra, Germany

In this section, it is necessary to discuss and examine the individual measurement series or measurement set-ups depicted in the figures in order to identify any potential anomalies regarding the propagation behavior of LoRa signals in underground mining operations.

### 8.1. Area 1 LoS

**Figure 8:**
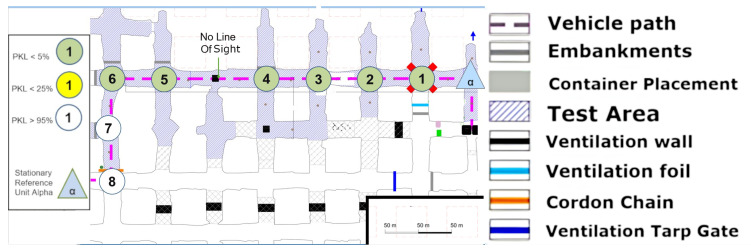
LoRa coverage measurement 1, LoS from the middle of the drift. The connectivity of the mobile unit, which is labeled with its position in Arabic numerals, is tested in relation to the stationary Alpha station. The Alpha station, which is represented by the triangle, remains stationary. The mobile unit is subsequently moved to the locations indicated, and the measurements are taken. In order to test the relative quality of the coverage in terms of its applicability for potential sensor set-up use cases, both the packet loss and the average dBm were taken. The average humidity was 42%, while the average temperature was 26 °C. It was found that neither temperature nor humidity had a measurable influence on these scales. The concrete measurement date can be found in the Appendix in Table A1.

The first set-up, depicted in Figure 8, represents an LoS set-up at a mining face. As expected, the direct path dominates. However, from point (4) onward, one of the fundamental aspects of this measurement series must already be addressed. LoS is lost very quickly due to the rapidly changing ground conditions in the drift, which do not remain constant along the assumed z-axis between points 4 and 5 (see Figure 8).

However, this level difference does not noticeably affect the signal quality. It can be assumed that the signal is reflected along the walls but above all along the floor and the roof. Due to the parabolic development of the drift with constant roof height, multiple reflections are the most obvious way of overcoming this topology. This implies that several reflections, at least three, would have to occur directly. The signal path can be best described by the term “bouncing”, as it requires multiple reflections with minimal loss of the signal itself. This initial observation leads to the conclusion that salt, in its current state within the mine, does not automatically absorb the signal in a manner analogous to a diffracting blackbody. Instead, a guiding effect seems to occur. To develop a model-based understanding of this phenomenon, it would be necessary to delve into the underlying physics and mathematics of wave–material interactions even further.

Due to the drift inclination between points 4 and 5 (as described above), the direct LoS to Alpha is lost at points 5 and 6. Point 7 is also in NLoS with Alpha, as illustrated in Figure 8, but there was a complete loss of signal from points (6) to (7). Upon closer examination of the map, the first assumption is that there is now a loss of LoS that cannot be overcome by multipathing via reflections or even bouncing. This is initially evidenced by the 100% signal drop over a few meters. If one were to assume a pure range problem, this would be accompanied by a large-scale path loss with a noticeable continuous loss, as predicted by the Friis model Equation (6). In other words, one would also observe an area that would only be moderately well covered. Based on the assumption that the observed multipath propagation results from signal reflections (commonly referred to as bouncing), it is crucial to understand why no comparable multipath propagation occurs between Points (6) and (7). In order for a 90° reflection to occur, as defined in Equation (4) and illustrated in Figure 2, the reflecting surface must support such behavior. However, the surface in this case consists of coarse and irregular salt structures, with a visible ventilation wall on the outer side, indicating a non-solid, rugged configuration. The front side is an open drift, suggesting that the consistency and topology of the salt medium may significantly impair reflective properties at this specific angle and position.

In the present constellation, only the reflected component of the electromagnetic wave is observed and relevant, as no signal was received at Point (7), thus excluding the possibility of transmission.

Furthermore, scattering-related phenomena such as multipath effects and bouncing are also considered subcategories of reflection in this context, and they are interpreted accordingly within the framework of the applied propagation model (see Figure 1).

### 8.2. Area 1 NLoS

**Figure 9:**
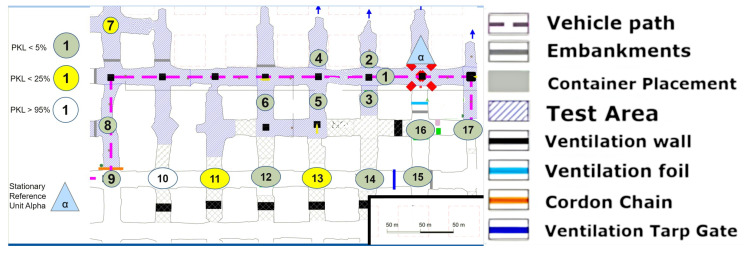
LoRa coverage measurement 2, NLoS outgoing from an excavation face. The connectivity of the mobile unit, which is labeled with its position in Arabic numerals, is tested in relation to the Alpha station. The triangularly depicted Alpha station remains stationary throughout the process. The mobile unit is then relocated to the indicated locations, and the measurements are taken. Of particular interest is the signal peak at position 12, which is a local peak. This peak is associated with the penetration capability of LoRa through the ventilation walls at this position. The location is a potash salt room-and-pillar mine, Werra, Germany, operated by the K+S group. The packet loss and average dBm were employed to assess the relative quality of the coverage in terms of its suitability for potential sensor set-up use cases. The average humidity was 43%, while the average temperature was 26 °C. Neither temperature nor humidity exerted a discernible influence on the scales in question. Please refer to the appendix table for the concrete measurement data.

**Figure 10:**
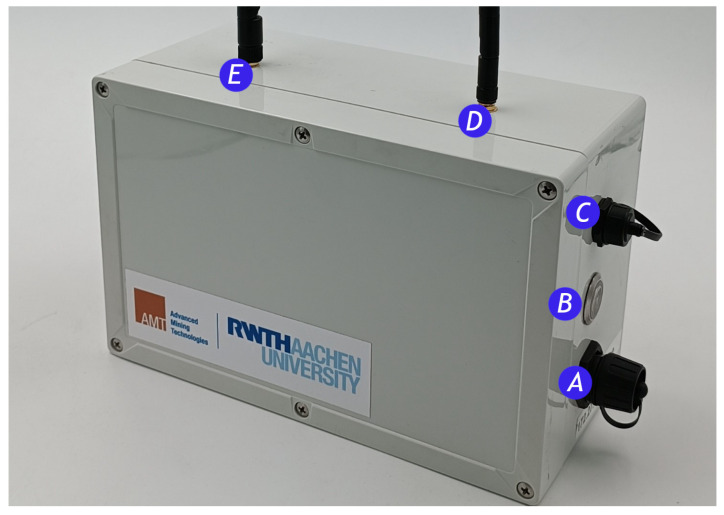
The “Walpurga” is a multifunctional research module unit capable of establishing LoRa networks among other connectivity options between itself and other devices. The unit is designed to be deployed with battery power for over a day under high computing use. It features a dust and water resistance rating (IP 66 rating). Components include Raspberry Pi, LoPy, GPS, battery, antenna, power circuitry. ***A***: Ethernet port; ***B***: LED power button; ***C***: USB-type micro-charging port; ***D***: GPS antenna for open-pit measurement capability; ***E***: omni-directional LoRa antenna.

The measurement in Figure 9 was conducted in the same area. In contrast to the previous measurement, this time, the Alpha station Figure 10 was not situated in the center of the section; instead, it was positioned in one of the mining syncline sections in order to facilitate the observation of the impact of varying NLoS.

The measurements were now always carried out with alternating drift offset. Consequently, the first measuring point (1) is no longer connected to the Alpha station via an LoS in the classical sense. A question from the previous measurement can now be answered directly by examining point (2) more closely. Between point (2) and Alpha, not only is there now a 90° turn, but a 90° turn must be made twice or at least at a radiated angle of 90° from the path itself. To ascertain the precise paths via ray tracing modeling, a corresponding simulation would be required here, for example, the dominant path model suggested by [28]. A concise overview of such “many ray” models as they are called, is given in [29]. However, it is evident that even within the assumed x-y plane, the LoRa signal has the potential to reflect at the inclination. Point (3) cannot be obtained even under a deviated assumed ray from the Alpha unit, as it is NLoS. An intermediate reflection would need to occur between the two walls, near measurement point (1). Therefore, multiple angular reflections are evident. It is important to note that points (4) and (5) must now be assessed in the same way. Furthermore, it should be emphasized that the quality of the signal remains unaltered; however, it is more probable that the number of necessary reflections at the joint will increase.

A noteworthy observation is made at point (7). This point is not only significantly further west than the Alpha station but also further north and more surrounded by the mineral. The position of Alpha in Figure 9 appears to be more optimal for enabling multiple reflections within the x-y plane. This assessment arises from the newly covered points in comparison to the set-up in Figure 8. However, the assumption that LoRa is not only conducive to single reflection but multiple reflections for multipath generation within the underground structure is confirmed.

The altered positioning of the Alpha module has now permitted the establishment of three additional coverage points in comparison to the configuration depicted in Figure 8. This represents a notable contrast, particularly when considering the typical installation of routers such as those utilized for Wi-Fi in urban or office environments. In such settings, it is generally preferable to locate these devices in open, spacious areas rather than in confined, secluded niches, as illustrated by this demonstration site.

Further points have been drawn on a new return path to the Alpha state; specifically, points (10) and (12) have been identified along with new insights. Point (10) is surrounded by two points on a cartographic straight line that have a much better connection to Alpha. But point (12) can be seen as a local maximum. It should be noted that none of the aforementioned points have an LoS, as they are shielded from the Alpha station by both a solid salt pillar and a ventilation wall. The question thus arises as to whether the points were reached by reflection or by penetration of the shielding material in front of Alpha. Although reflection has so far been considered the primary cause, the signal fluctuation on this straight line cannot be adequately explained by reflection. Assuming that the Alpha emission was reflected multiple times at a 90-degree angle into the straight line starting at point (15), the signal along the line from point (14) to (9) would become progressively weaker towards (9). Alternatively, if we consider the multipath from (15) as well as (9), there would either have to be signal connections everywhere along the line or a local minimum in the middle. Neither of these is the case, however. Therefore, it can be concluded that a penetration based on the different densities of the salt between these two lines to (12) or (10) must be taken into consideration, following the simple model outlined in Equation (2) and Figure 1 of the plane boundary transmission. This assumption is supported by the literature, which refers to the high signal budget of LoRa technology. The signal budget is a concept that enables the analysis of signal ranges within different materials related to a common metric. It is the proportional characteristic of the signal in comparison to the shielding quality of the material in the path direction. To illustrate, consider that lead has a high absorption in most cases, which results in the substantial reduction in the signal budget of a transmitter. In contrast, air or vacuum hardly ever absorbs signals.

### 8.3. Long Range Test

**Figure 11:**
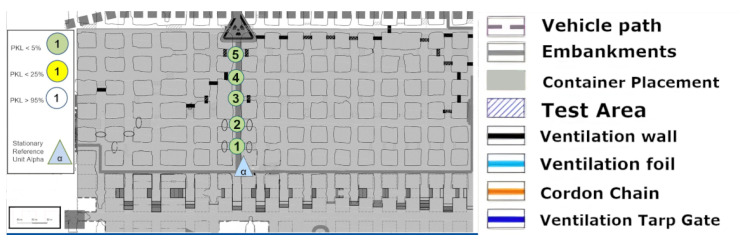
LoRa coverage measurement 3, long range with NLoS along the drift middle. The connectivity of the mobile unit, which is labeled with its position in Arabic numerals, is tested in relation to the Alpha station. The triangularly depicted Alpha station remains stationary throughout the process. The mobile unit is then relocated to the indicated locations, and the measurements are taken. Near point 5, LoS was lost due to elevation changes. It was not regained for the long-range test. The location is a salt room-and-pillar mine in Werra, Germany, operated by the K+S group. The packet loss and average dBm were employed to assess the relative quality of the coverage in terms of its suitability for potential sensor set-up use cases. The average humidity was 47%, while the average temperature was 27 °C. Neither temperature nor humidity exerted a discernible influence on the scales in question. Please refer to the appendix table for the concrete measurement data.

**Figure 12:**
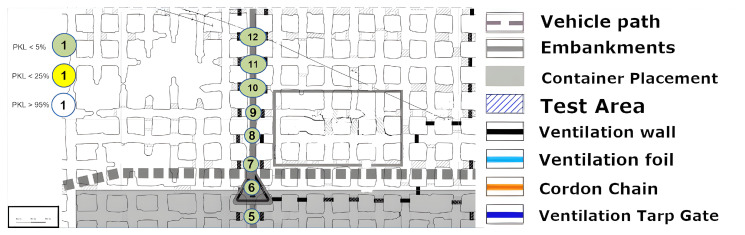
LoRa coverage measurement 3, continuation; long range with NLoS along the drift middle. The connectivity of the mobile unit, which is labeled with its position in Arabic numerals, is tested in relation to the Alpha station. The triangularly depicted Alpha station remains stationary throughout the process. The mobile unit is then relocated to the indicated locations, and the measurements are taken. Near point 5, LoS was lost due to elevation changes. It was not regained for the long-range test. The Alpha station remained as depicted in Figure 11. The location is a potash salt room-and-pillar mine, Werra, Germany, operated by the K+S group. The packet loss and average dBm were employed to assess the relative quality of the coverage in terms of its suitability for potential sensor set-up use cases. The average humidity was 47%, while the average temperature was 27 °C. Neither temperature nor humidity exerted a discernible influence on the scales in question. Please refer to the appendix table for the concrete measurement data.

**Figure 13:**
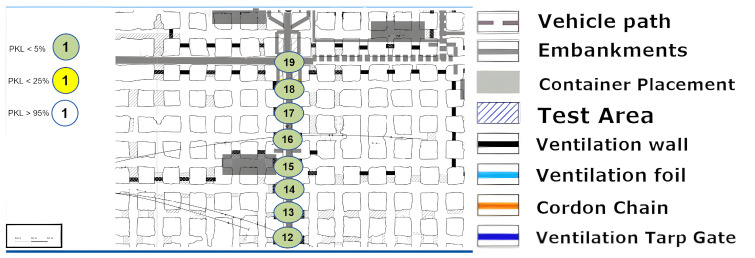
LoRa coverage measurement 3, continuation, continuation; long range with NLoS along the drift middle. The connectivity of the mobile unit, which is labeled with its position in Arabic numerals, is tested in relation to the Alpha station. The triangularly depicted Alpha station remains stationary throughout the process. The mobile unit is then relocated to the indicated locations, and the measurements are taken. The Alpha station remained, as depicted in Figure 11. The measurement stopped at 19 as the drift was blocked by a conveyor belt and a weather gate. The location is a potash salt room-and-pillar mine, Werra, Germany, operated by the K+S group. The packet loss and average dBm were employed to assess the relative quality of the coverage in terms of its suitability for potential sensor set-up use cases. The average humidity was 47%, while the average temperature was 27 °C. Neither temperature nor humidity exerted a discernible influence on the scales in question. Please refer to the appendix table for the concrete measurement data.

The discussion of Figure 11, Figure 12 and Figure 13 can be most effectively addressed in a joint paragraph. This investigation sought to assess the long-range capacity of LoRa in the underground environment. In the context of wireless technologies, there is no clear-cut line drawn as to when a technology is considered to be long-range. Yet at radiation powers of less than one kilowatt, several hundred meters are considered as a long-range portion of the radiation, and a kilometer range clearly stands for this characteristic of the technology. This is according to the developer of LoRa SemTech in direct comparison with other frequency-shift keying technologies such as RFID systems [24]. Hence, the most direct and uninterrupted route in the x-y plane was selected from the area. An effective limit was to be identified here. However, a physical limit at the end of this lengthy stretch over kilometers was the determining factor. Despite point (19) demonstrating optimal signal propagation, the physical path was cut off via a gate and material rerouting unit. A retrospective analysis in Figure 11 shows that the LoS in the z-axis was no longer available from points (4)–(5). The clear difference between these two measurement areas is that in Figure 11 and Figure 12, the height differences do not occur in a single step. Instead, over the entire distance, the floor’s height varies. Therefore, for a true straight beam from the Alpha station, there would be several hundred meters of solid salt to penetrate. This provides clear confirmation that the LoRa signal has the possibility to follow the general topology of the tube-like drift. Furthermore, the bouncing, or reflection, of the signal must occur with great frequency, as the change in altitude at a fixed roof height is achieved by a greater number of reflections. Consequently, the signal must be reflected or bounced multiple times to reach point (19). Nevertheless, these reflections appear to have a high degree of signal retention, which allows them to remain in the measurement regime. This is an extreme case for straight and long unconfined distances, with minimal packet or signal quality loss. It should be noted that the measurement shown in Figure 11 indicates that the path must not be too steep and must rise again; otherwise, the signal may not follow the narrow path. In this case, the signal may be reflected backward as in a parabolic mirror.

**Figure 14:**
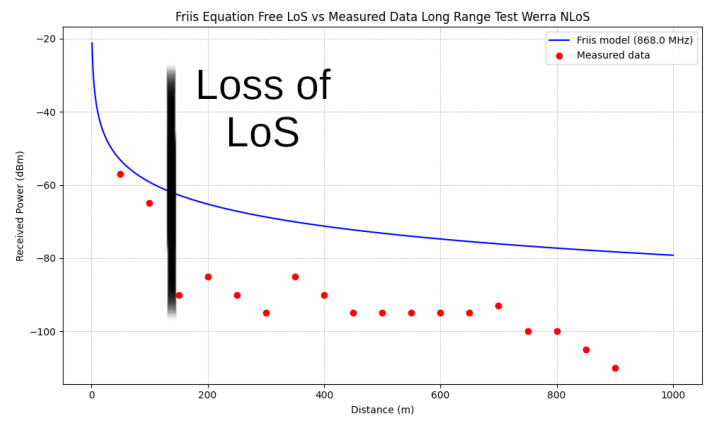
Friis Equation free LoS (in blue) vs. measured data (in red) long-range test Werra NLoS. The measurement points are significantly below the expected values after the loss of LoS. A logarithmic trend can be seen, but the standard Friis Equation only applies in free LoS situations. One effective modification could be to introduce relative distances by summing the reflected signal path lengths, which are greater than the straight LoS distances.

A 2D graphical representation can be used effectively with the long-range path length as the x-axis. In the Figure 14, the expected course of the signal loss according to the Friis Equation (6) was plotted with the measured data.

Although the values are significantly lower in signal strength than predicted by the model, the logarithmic trend can be recognized. In particular, there is a sudden deviation once the LoS is lost. However, as already described in the Friis Equation (6), the application of this equation for simple LoS ratios can be calculated without reflections, multipathing, or obstacles of any kind.

A hard cutoff is often applied at the LoS. This was not carried out here in order to clarify the logarithmic progression behind the LoS line. It can be seen that a modification of the model or the input parameters must be made in order to continue using this basic model. Here, it can be assumed as a simple modification that for the distance, instead of assuming a real straight line, the total length of the reflected rays from the bouncing adds up to significantly longer paths, resulting in a relative distance, which in turn can serve as input for the Friss model.

### 8.4. Excavation Face near LoS

**Figure 15:**
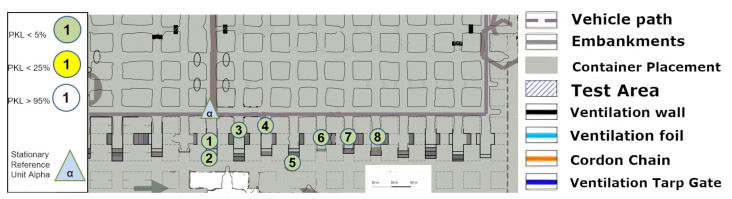
LoRa coverage measurement 4, part 1 of 3; LoRa coverage at the active excavation face to establish possible network expectations at logistically non-stationary areas of interest. The connectivity of the mobile unit, which is labeled with its position in Arabic numerals, is tested in relation to the Alpha station. The triangularly depicted Alpha station remains stationary throughout the process. The mobile unit is then relocated to the indicated locations, and the measurements are taken. The location is a potash salt room-and-pillar mine, Werra, Germany, operated by the K+S group. The packet loss and average dBm were employed to assess the relative quality of the coverage in terms of its suitability for potential sensor set-up use cases. The average humidity was 50%, while the average temperature was 27 °C. Neither temperature nor humidity exerted a discernible influence on the scales in question. Please refer to the appendix table for the concrete measurement data.

**Figure 16:**
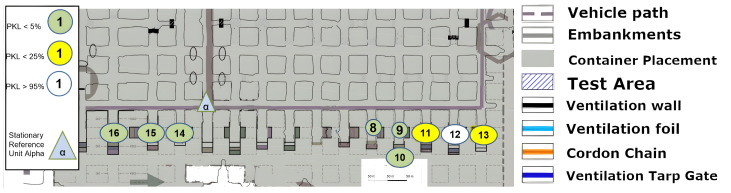
LoRa coverage measurement 4, part 2 of 3; LoRa coverage at the active excavation face to establish possible network expectations at logistically non-stationary areas of interest. The connectivity of the mobile unit, which is labeled with its position in Arabic numerals, is tested in relation to the Alpha station. The triangularly depicted Alpha station remains stationary throughout the process. The mobile unit is then relocated to the indicated locations, and the measurements are taken. The location is a potash salt room-and-pillar mine, Werra, Germany, operated by the K+S group. The packet loss and average dBm were employed to assess the relative quality of the coverage in terms of its suitability for potential sensor set-up use cases. The average humidity was 50%, while the average temperature was 27 °C. Neither temperature nor humidity exerted a discernible influence on the scales in question. Please refer to the appendix table for the concrete measurement data.

**Figure 17:**
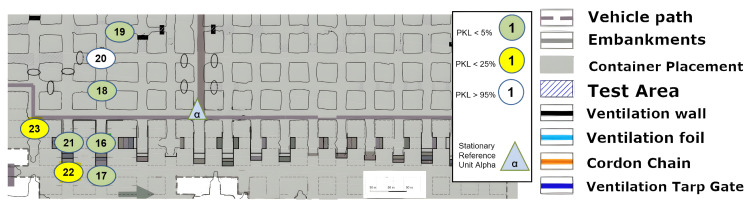
LoRa coverage measurement 4, part 3 of 3; LoRa coverage at the active excavation face to establish possible network expectations at logistically non-stationary areas of interest. The connectivity of the mobile unit, which is labeled with its position in Arabic numerals, is tested in relation to the Alpha station. The triangularly depicted Alpha station remains stationary throughout the process. The mobile unit is then relocated to the indicated locations, and the measurements are taken. The location is a potash salt room-and-pillar mine, Werra, Germany, operated by the K+S group. The packet loss and average dBm were employed to assess the relative quality of the coverage in terms of its suitability for potential sensor set-up use cases. The average humidity was 50%, while the average temperature was 27 °C. Neither temperature nor humidity exerted a discernible influence on the scales in question. Please refer to the appendix table for the concrete measurement data.

The measurement that was graphically split on Figure 15, Figure 16 and Figure 17 is now to be considered. The signal was to be tested for the coverage of operationally relevant mining sections. The LoS was not given, but the signal reached almost all mining faces, as can be seen. Once more, a reflection must allow multipathing, as the local minimum or coverage hole at (12) cannot be explained by a non-applicable reflection environment. Furthermore, a signal with large-scale path loss does not improve again if one goes a little further to point (13). Points (18), (19) and (20) illustrate the complex structure of a coverage prediction. Even under the assumption that only reflections or only penetration of ventilation airflow would allow the coverage of these points, numerous pathways must be considered or calculated, as a variety of effects must be accounted for. However, it appears that at these points, even the combination of penetration and reflection over multiple pathways must be calculated and included.

## 9. Discussion on Propagation Behavior in a Sandstone Mine Kvarntrop, Sweden

In order to facilitate a meaningful comparison, the aforementioned LoS, NLoS, and long-distance measurements were taken again in a new environment. The hardware was set up in Sweden, in the vicinity of the city of Örebro. The test mine Kvarntrop, which was previously a sandstone mine, offered an extremely similar wall-and-rock topology on a macroscopic level, as well as a superimposed structure that resembled the structure in the potash salt mine in Werra, Germany. In essence, the struts were relatively straight and horizontal, connected by almost completely straight turns.

### 9.1. Test Area LoS

**Figure 18:**
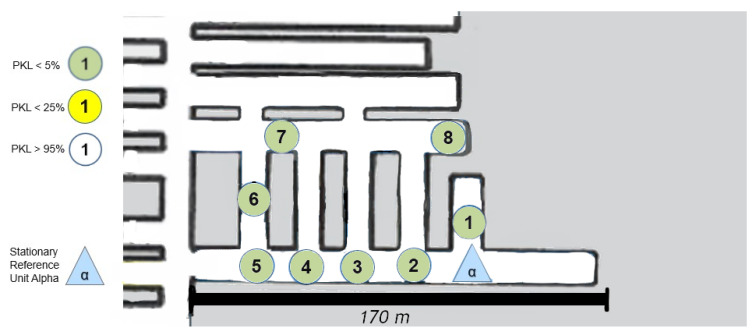
LoRa coverage LoS measurement 5, LoRa coverage test mine in Sweden. The connectivity of the mobile unit, which is labeled with its position in Arabic numerals, is tested in relation to the Alpha station. The triangularly depicted Alpha station remains stationary throughout the process. The mobile unit is then relocated to the indicated locations, and the measurements are taken. The location is the Kvarntorp test mine near Örebro, Sweden, used by Epiroc, among others. It was primarily a sandstone mine.

Figure 18 presents an image that can be directly compared with the one in Figure 8 in terms of its shape and size. The propagation of the signal can be attributed to a number of additional angles and reflections.

### 9.2. Test Area NLoS

**Figure 19:**
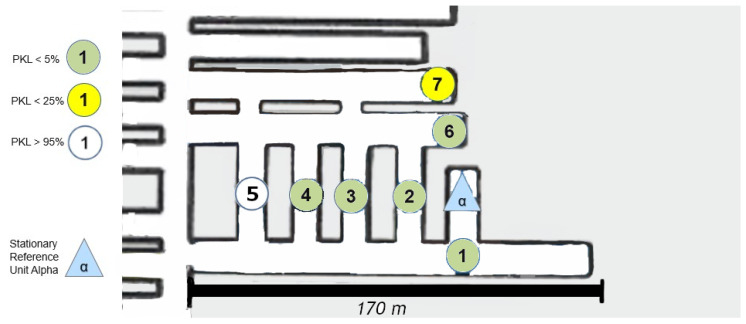
LoRa propagation at Kvarntorp next to a muck pile, LoRa coverage measurement 6, LoRa Coverage test mine in Sweden. The connectivity of the mobile unit, which is labeled with its position in Arabic numerals, is tested in relation to the Alpha station. The triangularly depicted Alpha station remains stationary throughout the process. The mobile unit is then relocated to the indicated locations, and the measurements are taken. The location is the Kvarntorp test mine near Örebro, Sweden, used by Epiroc, among others. It was primarily a sandstone mine. The packet loss and average dBm were employed to assess the relative quality of the coverage in terms of its suitability for potential sensor set-up use cases. Please refer to the appendix table for the concrete measurement data.

The assumption that reflection is once again the primary factor in the signal propagation assessment is demonstrated in Figure 19. Point (5) is not covered, despite the necessity of a reflection or multiple reflections for point 4. Consequently, point (7) is almost impossible to describe by reflection, whereas point (6) is still perfectly covered. If a reflection were to extend from point (5) to point (7), point (5) would have to be covered, by logical necessity. It can therefore be concluded that (6) can be reached via reflection without significant signal loss, whereas (7) can still be reached by penetrating the face material via the signal budget and thus reduced signal interception.

### 9.3. Kvarntorp Long Range

**Figure 20:**
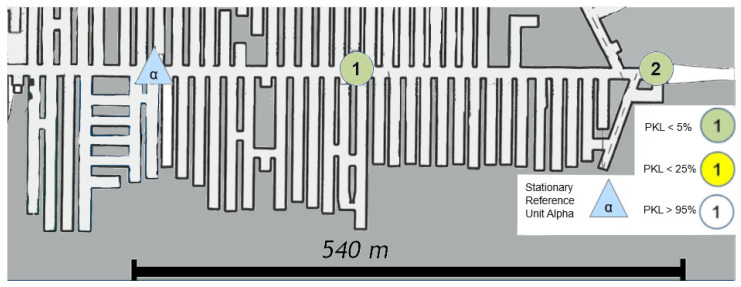
LoRa long-range measurement 7; LoRa coverage test mine in Sweden. The connectivity of the mobile unit, which is labeled with its position in Arabic numerals, is tested in relation to the Alpha station. The triangularly depicted Alpha station remains stationary throughout the process. The mobile unit is then relocated to the indicated locations, and the measurements are taken. The location is the Kvarntorp test mine near Örebro, Sweden, used by Epiroc, among others. It was primarily a sandstone mine. The packet loss and average dBm were employed to assess the relative quality of the coverage in terms of its suitability for potential sensor set-up use cases.

A description of the results in Figure 20 also shows that LoRa can cope with changes in altitude on a straight line, even in a sandstone mine. However, in this instance, the altitude is just under 800 m, even without LoS.

In summary, the following primary effects can be assumed from the measurements: It can be postulated that LoRa can be ideally reflected by materials such as salt and rock. Below a certain thickness of the material, penetration is possible, but often with a signal that decreases in quality. This ideal reflection allows the LoRa signal to be guided far, similar to a waveguide in optical physics, with idealized transmission. In the context of an underground mine, one can understand the principle of a waveguide as the name implies. The surrounding medium boundary prevents the equal expansion of the signal in space but directs or guides it on a path determined by the medium’s boundaries. A deep analysis of this principle from the time of Maxwell and Rayleigh to the present is given in [30]. From a certain degree of complexity of the area, several paths and both effects can coincide, representing high complexity in a prediction model. The wavelength from the comparison with Wi-Fi or 5G in the areas is also a factor. The topology of the walls and the structure of the drift represent physical influencing factors that result in different environmental parameters than those observed in urban or office-like environments.

### 9.4. Correlating the Propagation Concepts

A broad spectrum of propagation theory and practice was established in Section 3 and Section 4, both to enable a structured interpretation of the measured data and to provide readers with an overview of the analytical tools applied in this work. This section revisits the previously introduced theoretical aspects in the context of the actual measurement results, highlighting which theories were supported or limited by empirical findings.

Starting with the Maxwell equations, these remain fundamentally valid in the operational regimes considered here, as expected. Via appropriate limit-taking for high frequencies, the ray-based models (geometrical optics) used throughout this paper can be derived. These form the theoretical basis for the observed directional signal behaviors across long distances and multiple tunnel junctions.

Scattering and refraction were repeatedly used in Section 8.1 and onward to explain specific signal anomalies and propagation phenomena. These mechanisms proved essential for understanding multipath components and unexpected reception in NLoS regions, especially around pillar edges and drift junctions.

Transmission and penetration were also discussed; however, as outlined previously, they must be conceptually separated from diffraction. While some form of diffraction may theoretically occur in the presence of loosely compacted materials, such as backfill or crushed rock, the lack of homogeneity and the absence of fully solid interfaces in these regions limit meaningful diffraction analysis there. In contrast, the effects of diffraction on solid, high-density walls were expected to be more relevant for the plausible explanation of certain NLOS reception events. Still, at the power levels used in this study, the solid rock walls were not measurably transmissive or diffraction-inducing.

Absorption, a factor of increasing importance at higher frequencies, was also considered. As noted earlier, radiation losses due to air absorption were found to be negligible within the spatial and frequency ranges of this study, and can be safely omitted for conceptual network designs operating under similar conditions.

A final but important point is the deliberate exclusion of shadowing effects caused by large machinery or operational equipment. This choice was made to allow for a baseline measurement of the propagation environment. Shadowing in this study was thus primarily caused by the surrounding structural pillars, which served as dominant obstructions. Interestingly, several instances of apparent NLOS communication—where shadowing should have occurred—were instead interpreted through the lens of scattering and reflection, providing a consistent explanation for otherwise unexpected reception quality.

Among the macroscopic concepts of radio wave propagation, the most relevant in the context of this study is their mitigation potential in the design of underground communication networks. These high-level effects influence the robustness and coverage stability of a deployed system, and understanding them enables better-informed network planning in mining environments.

A central theoretical and practical focus of this work is the spatio-temporal variation of the channel, which represents the combined impact of microscopic and time-dependent influences on signal behavior. This analysis emphasized such variations as key limiting factors for real-world deployment, and the measurements were deliberately designed to observe these effects under controlled, static conditions.

Specifically, temporal variations, such as those caused by equipment motion, personnel movement, or dynamic repositioning of nodes, were explicitly minimized or excluded from the set-up. This approach ensured the capture of baseline propagation characteristics, which would otherwise be masked or distorted by temporal artifacts. However, the implications of these variations remain crucial for future studies that aim to integrate the presented results into active network scenarios or adaptive network reconfigurations.

The large-scale path loss was explicitly investigated in Section 8.3, capturing the general attenuation trend over distance, particularly across multiple tunnel turns and extended drift segments.

As previously discussed, delay spread plays a significant role in the measurements due to the highly reflective mineral walls. In long-distance measurements, multipath propagation and delayed signal copies should not be ignored. The simplistic single-ray idealization becomes insufficient in such scenarios, especially in long and geometrically complex drifts, where reflections and scattering dominate signal behavior.

Regarding frequency dependence, LoRa’s sub-GHz operation reduces susceptibility to many environmental resonances, such as humidity- or moisture-related absorption, which are more pronounced at higher frequencies. In this sense, frequency-dependent effects in this study were found to be minimal to negligible, further supporting the choice of LoRa for underground applications.

Lastly, considerations of noise and co-channel interference are particularly favorable in the underground mining context. As expected, only the locally deployed networks were detectable during measurements, allowing for a clean analysis free of external interference. This relative spectral isolation is a notable advantage for network design, allowing system interactions to be modeled based solely on in-network behavior, which should be leveraged when designing robust and interference-resilient underground communication systems.

### 9.5. Derivation of Bouncing and Relative Path Length

**Figure 21:**
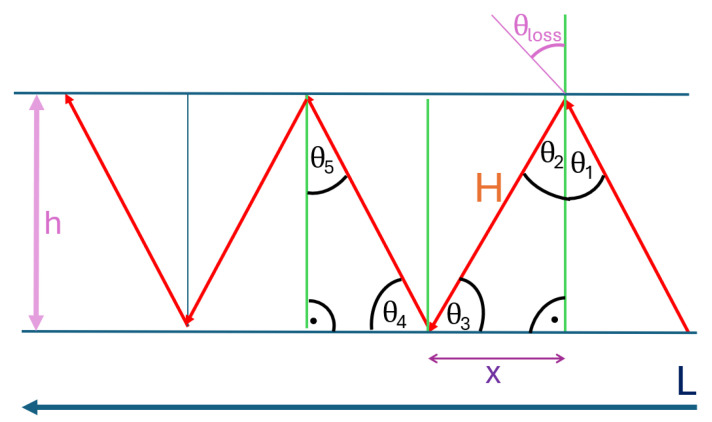
A 2D sketch of a bouncing ray in a straight drift of length L, with a constant height h. For simplicity, only the first transmitted ray is drawn.

At this point, a mathematical description is provided with the aim of deriving the bouncing effect resulting in a waveguide-like behavior. The simplest case based on a straight drift with constant height is considered; see Figure 21. The results found are compared with the proposed modification for the Friis model in Figure 14. The generalization of the derivation to any meaningful drift shape is only sketched here.

- Total drift length: *L*;
- $\theta_{1,\ldots ,m}$: incident and reflected angles;
- $\theta_{1}$ is assumed to be given;
- *h*: drift height
- *x*: partial section of *L* framed by two reflections;
- *H*: hypotenuse;
- $\theta_{\mathrm{loss}}$: transmission angle according to Snell’s Law Equation (4).

The goal is to derive a general relation for all $\theta_{1},\ldots ,\theta_{n}$, and determine the relative path length $L_{R}$ based on a summation over *H*. Only Snell’s Law 4 and basic trigonometry are used for the 2D straight drift case.

Working within the first triangle in Figure 21 formed by *x*, *h*, and the angle $\theta_{2}=\theta_{1}$ via Equation (4),

$$\tan\left(\theta_{1}\right)=\frac{x}{h}\;\Rightarrow \;x=\tan\left(\theta_{1}\right)\cdot h$$

$$H=\frac{h}{\cos(\theta_{1})}$$

Angle $\theta_{3}$ can be derived via $\tan(\theta_{1})$ or simply over a 180° sum in a triangle, also reading π in radians:

$$\theta_{3}=\arctan\left(\frac{1}{\tan(\theta_{1})}\right)=180^{\circ }-90^{\circ }-\theta_{1}=90^{\circ }-\theta_{1}=\pi -\theta_{1}$$

Thus, by symmetry and Snell’s law,

$$\theta_{3}=\theta_{4},\;\theta_{5}=\pi -\theta_{3}=\theta_{1}$$

Assuming the total length of the drift is L=m·x, where *m* is the number of reflections divided by two, it follows that

$$m=\frac{L}{x}=\frac{L}{\tan(\theta_{1})\cdot h}$$

Now, the desired relative path length $L_{R}$ based on the summation over *H*:

$$L_{R}=m\cdot H=m\cdot \frac{h}{\cos(\theta_{1})}=\frac{L}{\tan(\theta_{1})\cdot h}\cdot \frac{h}{\cos(\theta_{1})}$$

$$L_{R}=\frac{L}{\tan\left(\theta_{1}\right)\cdot \cos\left(\theta_{1}\right)}=\frac{L}{\frac{\sin(\theta_{1})}{\cos(\theta_{1})}\cdot \cos\left(\theta_{1}\right)}=\frac{L}{\sin(\theta_{1})}$$

**Conclusion:** Straight drifts result in relative lengths $L_{R}=\frac{L}{\sin(\theta_{1})}$ fitting the signal loss in Figure 14.

A brief outline of the steps required to generalize this result is as follows: The first point would be a summation and average over all emitted angles $\theta_{1}$. On top of that, to get to three dimensions, one would then have to calculate an integral over the contour of the drift at each point. This could be approximated by radial integrals but only in the first order. An important assumption is that the height *h* remains constant; this can also be kept in the first order because the height is rather stable underground to guarantee vehicle use without problems and not to loosen too much surrounding material. However, as already described in the discussion, the assumption of straight drift is not valid for more than the distance of one or two measuring points. Therefore, one would either have to create a segmentation in individual sections of the length of two measuring points or create a continuous function for the curve of the drift. These are then to be applied in such a way that they are reflected in the length *L* and above all in a modification of the angle θ. For narrow angles, the approximation can be assumed to be implemented using segmented drift elements.

## 10. Conclusions

This paper presents and discusses the findings of a study on LoRa propagation in real underground mining environments of the room and pillar type. It focuses on the technical set-up, the relevance of network analysis for the mine of the future, and prospects for future propagation models. Three main propagation set-ups were demonstrated and explained, as well as the novel and surprising effects observed in terms of LoRa’s propagation behavior in these environments. The findings revealed that the signal exhibited a bouncing behavior, whereby it was reflected on the walls, roof, and floor. This phenomenon was termed a “waveguide-like” behavior due to LoRa’s ability to follow the deviating nature of the mining drifts as they follow the mineral deposits. This signal-bouncing behavior was also observed by P. Branch. [10], which was attributed to steel floor plates present in the drift corridors. In the present study, similar bouncing effects were observed independently in both measurement locations, despite the absence of steel flooring. This recurrence suggests that the observed reflections may not be solely due to human-made elements like steel plates but instead may be significantly influenced by the surrounding geological material, adding a new perspective to the interpretation of such effects.

Furthermore, LoRa’s capability to penetrate solid minerals like salt and rock lined with sandstone deposits demonstrated its effectiveness over ranges of several kilometers underground, following the aforementioned waveguide principle.

Comparative studies of two different mines have indicated that the observed effects can be transferred to other mine sites. The uniqueness of the mining environment opens up new avenues for modeling signal behavior. These findings highlight the importance of rethinking network structure and signal behavior models for applications in underground mining. The waveguide-like effect is a significant benefit that should be fully analyzed and utilized. Furthermore, the interactions of LoRa with other networks, whether already in use or newly installed in a mine, require further investigation.

Future research should explore the influence of different minerals, mine topologies, structures, and frequencies on signal behavior. Additionally, there is a need to investigate the possibilities of LoRa’s interaction with other networks and its use-case-oriented applications based on these findings. An important extension to a future measurement campaign would be an accompanying spectrum analyzer to capture the SNR and RSSI values for all points.

The results of the measurements and discussions clearly imply that the network structure and behavior modeling need to be reconsidered for applications in underground mining. A key limitation of LoRa, as with most wireless communication systems operating underground, is the limited material penetration, especially through dense mineral structures. Consequently, the deployment of repeaters or mesh-based network architectures becomes essential to maintain connectivity over extended areas.

Additionally, the data rate achievable at the employed sub-GHz frequencies and modulation schemes represents another constraining factor. These limitations confine potential use cases primarily to applications such as machine condition monitoring, heartbeat signaling, and non-time-critical data relaying, where low data throughput and higher latency can be tolerated.

This does not necessarily mean that underground mining is more challenging than urban or office-based networking. The measurements and the waveguide-like effect present significant benefits that need to be fully analyzed and utilized. As stated in this paper and in many conferences, the mining sector is developing toward the digital mine of the future. Networks play a pivotal role in this endeavor. The measurement series outlined in this paper serves to illustrate that networking underground is feasible with LoRa, yet its behavior is as distinctive compared to urban settings, as the mining environment itself is a research field and part of the global economy.

## Data Availability

Data are contained within the article.

## Appendix A. Tables of Measurement Results

**Table A1:** Measurement results of first LoS set-up, testing area, potash salt, K+S group in the Werra mine, Germany, as graphically depicted in Figure 8.

Label	Averg. dBm	% PKG Loss	% Humidity	T in °C
1	−55	0/0/0	43	26
2	−53	0/0/0	45	26
3	−67	0/0/0	42	26
4 ^1^	−70	0/0/0	44	26
5	−83	0/0/0	42	27
6	−96	0/0/0	40	27
7	NaN	100/100/100	39	27
8	NaN	100/100/100	42	27

^1^ Loss of Sight. The data was collected in real time and verified on-site. It should be noted that the humidity and temperature remained constant throughout the experiment. A review of standard literature indicates that the wavelength of the LoRa signal should not experience any significant or measurable influences in these scales for the given set-up and applications [31]. The test was conducted in a testing and training area as part of the overall chosen mining sector at a depth of approximately 800 m.

**Table A2:** Measurement results of NLoS set-up, testing area, potash salt, K+S group in the Werra mine, Germany, as graphically depicted in Figure 9.

Label	Averg. dBm	% PKG Loss	% Humidity	T in °C
1	−70	0/0/0	42	26
2	−78	0/0/0	41	26
3	−90	0/0/0	42	26
4	−100	0/0/0	42	26
5	−100	0/0/0	41	26
6	−100	0/0/0	44	26
7	−110	26/18/10	49	26
8	−110	0/0/0	45	26
9	−110	2/0/0	44	26
10	−110	48/92/90	41	26
11	−110	4/30/0	44	26
12	−110	0/0/2	45	26
13	−110	14/6/22	43	26
14	−100	0/0/2	42	26
15	−65	0/0/0	43	26
16	−60	0/0/0	43	26
17	−85	0/0/0	42	26

The data was collected in real time and verified on-site. It should be noted that the humidity and temperature remained constant throughout the experiment. A review of standard literature indicates that the wavelength of the LoRa signal should not experience any significant or measurable influences in these scales for the given set-up and applications [31]. The test was conducted in a testing and training area as part of the overall chosen mining sector at a depth of approximately 800 m.

**Table A3:** Measurement results of the long-range set-up, training area, potash salt, K+S group in the Werra mine, Germany, as graphically depicted in Figure 11, Figure 12 and Figure 13.

Label	Averg. dBm	% PKG Loss	% Humidity	T in °C
1	−57	0/0/0	47	27
2	−65	0/0/0	47	27
3	−90	0/0/0	47	27
4	−85	0/0/0	51	27
5	−90	0/0/0	52	27
6	−95	0/0/2	57	27
7	−95	2/0/0	52	26
8	−85	0/0/0	53	27
9	−90	0/0/0	44	26
10	−95	0/0/0	41	26
11	−95	0/0/0	44	26
12	−95	0/0/2	45	26
13	−95	0/2/0	43	28
14	−95	0/0/0	42	28
15	−93	0/0/0	43	27
16	−100	0/0/0	43	27
17	−100	0/2/0	42	27
18	−105	4/0/0	44	27
19	−110	0/7/6	44	27

The data was collected in real time and verified on-site. It should be noted that the humidity and temperature remained constant throughout the experiment. A review of standard literature indicates that the wavelength of the LoRa signal should not experience any significant or measurable influences in these scales for the given set-up and applications [31]. The test was conducted in the southern training area as part of the overall chosen mining sector at a depth of approximately 800 m.

**Table A4:** Measurement results of the excavation face, training area, potash salt, K+S group in the Werra mine, Germany. Graphically depicted in Figure 15, Figure 16 and Figure 17.

Label	Averg. dBm	% PKG Loss	% Humidity	T in °C
1	−55	0/0/0	47	27
2	−55	0/0/0	47	27
3	−80	0/0/0	47	27
4	−85	0/0/0	47	26
5	−100	2/0/0	47	27
6	−100	0/0/0	47	27
7	−105	0/0/0	46	27
8	−100	0/0/0	47	27
9	−100	0/0/0	47	27
10	−110	0/0/0	47	27
11	−115	38/16/6	47	27
12	*NaN*	100/100/100	47	27
13	−115	12/0/2	46	27
14	−85	0/0/0	47	26
15	−100	0/0/0	47	27
16	−105	0/2/0	47	27
17	−100	0/0/0	46	27
18	−105	0/0/0	47	27
19	−105	0/0/0	47	27
20	*NaN*	100/100/100	47	27
21	−105	0/0/0	47	27
22	−115	38/58/38	47	27
23	−115	26/39/40	47	26

The data was collected in real time and verified on-site. It should be noted that the humidity and temperature remained constant throughout the experiment. A review of standard literature indicates that the wavelength of the LoRa signal should not experience any significant or measurable influences in these scales for the given set-up and applications [31]. The test was conducted in the southern training area as part of the overall chosen mining sector at a depth of approximately 800 m.

**Table A5:** Measurement results of the LoS test in the experimental mining area, rock, Epiroc, in Kvarntorp near Örebro, Sweden.

Label	Averg. dBm	% PKG Loss
1	−48	0/0/0
2	−65	0/0/0
3	−64	0/0/0
4	−79	0/2/0
5	−77	0/0/0
6	−93	0/0/0
7	−103	2/0/0
8	−80	0/0/0

The data was collected and verified on-site. It should be noted that the humidity and temperature remained constant throughout the test. A review of the relevant literature indicates that the wavelength of the LoRa signal should not experience any significant or measurable influences in these scales for the given set-up and applications [31]. The test was conducted in the experiment area as part of the shared testing mine at a depth of approximately 50 m. Depicted graphically in Figure 18.

**Table A6:** Measurement results of the NLoS test in the experimental mining area, rock, Epiroc, in Kvarntorp near Örebro, Sweden.

Label	Averg. dBm	% PKG Loss
1	−64	0/0/0
2	−84	0/0/0
3	−110	18/0/0
4	−109	0/0/0
5	xx	96/100/100
6	−107	0/4/0
7	−114	72/18/22

The data was collected and verified on site. It should be noted that the humidity and temperature remained constant throughout the test. A review of the relevant literature indicates that the wavelength of the LoRa signal should not experience any significant or measurable influences in these scales for the given set-up and applications [31]. The test was conducted in the experiment area as part of the shared testing mine at a depth of approximately 50 m. Depicted graphically in Figure 19.

**Table A7:** Measurement results of the long-range test in the experimental mining area, rock, Epiroc, in Kvarntorp near Örebro, Sweden.

Label	averg. dBm	% PKG Loss
1	−85	0/0/0
2	−85	0/0/0
3	−74	0/0/0

The data was collected and verified on-site. It should be noted that the humidity and temperature remained constant throughout the test. A review of the relevant literature indicates that the wavelength of the LoRa signal should not experience any significant or measurable influences in these scales for the given set-up and applications [31]. The test was conducted in the experiment area as part of the shared testing mine at a depth of approximately 50 m. Depicted graphically in Figure 20.
